# Photodynamic and Photothermal Therapy of Hepatocellular Carcinoma

**DOI:** 10.3389/fonc.2021.787780

**Published:** 2021-12-07

**Authors:** Zhe Fan, Chengjun Zhuang, Shuang Wang, Yewei Zhang

**Affiliations:** ^1^ Department of General Surgery, the Third People’s Hospital of Dalian, Dalian Medical University, Dalian, China; ^2^ Department of Central Laboratory, the Third People’s Hospital of Dalian, Dalian Medical University, Dalian, China; ^3^ Department of Critical Care Medicine, The Second Affiliated Hospital of Dalian Medical University, Dalian, China; ^4^ Department of Endocrinology, The Second Affiliated Hospital of Dalian Medical University, Dalian, China; ^5^ Department of Hepatobiliary and Pancreatic Surgery, The Second Affiliated Hospital of Nanjing Medical University, Nanjing, China

**Keywords:** photodynamic therapy, photothermal therapy, hepatocellular carcinoma, treatment, review

## Abstract

Hepatocellular carcinoma (HCC) is the most common primary liver tumor. It is ranked the sixth most common neoplasm and the third most common cause of cancer mortality. At present, the most common treatment for HCC is surgery, but the 5-year recurrence rates are still high. Patients with early stage HCC with few nodules can be treated with resection or radiofrequency ablation (RFA); while for multinodular HCC, transarterial chemoembolization (TACE) has been the first-line treatment. In recent years, based on medical engineering cooperation, nanotechnology has been increasingly applied to the treatment of cancer. Photodynamic therapy and photothermal therapy are effective for cancer. This paper summarizes the latest progress of photodynamic therapy and photothermal therapy for HCC, with the aim of providing new ideas for the treatment of HCC.

## Introduction

Cancer is the second most common cause of death among all diseases (1). Hepatocellular carcinoma (HCC) is a common digestive system tumor and the sixth most common type of cancer worldwide (2). Treatment includes radical surgery (3), molecular targeted therapies (4) and neoadjuvant therapy (5). Although progress has been made in the treatment of HCC, the prognosis of HCC patients is still poor and the 5-year survival rate is only about 18% (6). Therefore, new treatment methods are urgently needed to change this situation.

**Figure 1:**
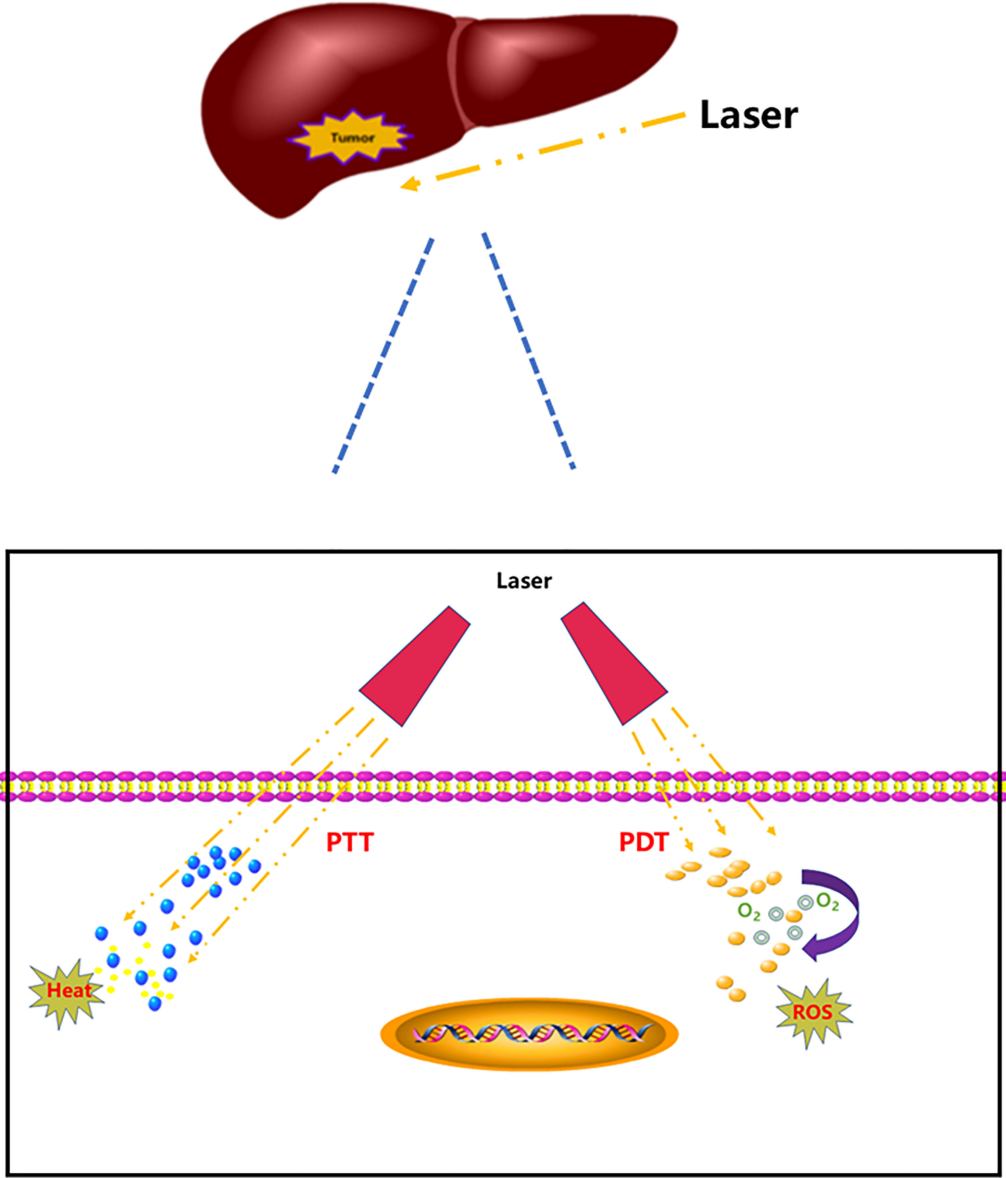
PTT and PDT for HCC.

The toxicity and adverse effects of antitumor drugs have led researchers to seek new tumor treatment strategies (7) and photothermal therapy (PTT) and photodynamic therapy (PDT) have gradually emerged because of their specific spatial selectivity and lower invasiveness and initial resistance (8–10). PTT is a tumor treatment strategy that utilizes photothermal agents to induce thermal energy by laser. Absorbed light energy can be transformed into heat energy to achieve thermal ablation of tumor cells; therefore, tumors can be killed in the high temperature environment (11–13). PDT takes advantage of the active metabolism of tumor tissue; whereby non-toxic photosensitizers accumulate in tumor tissue after injection. When the tumor tissue is irradiated with harmless visible light, the activated photosensitizer transfers its energy to surrounding intracellular oxygen that forms reactive oxygen species (ROS), which specifically destroy the tumor cells and neovascularization (14–17) (**Figure 1**).

**Figure 2:**
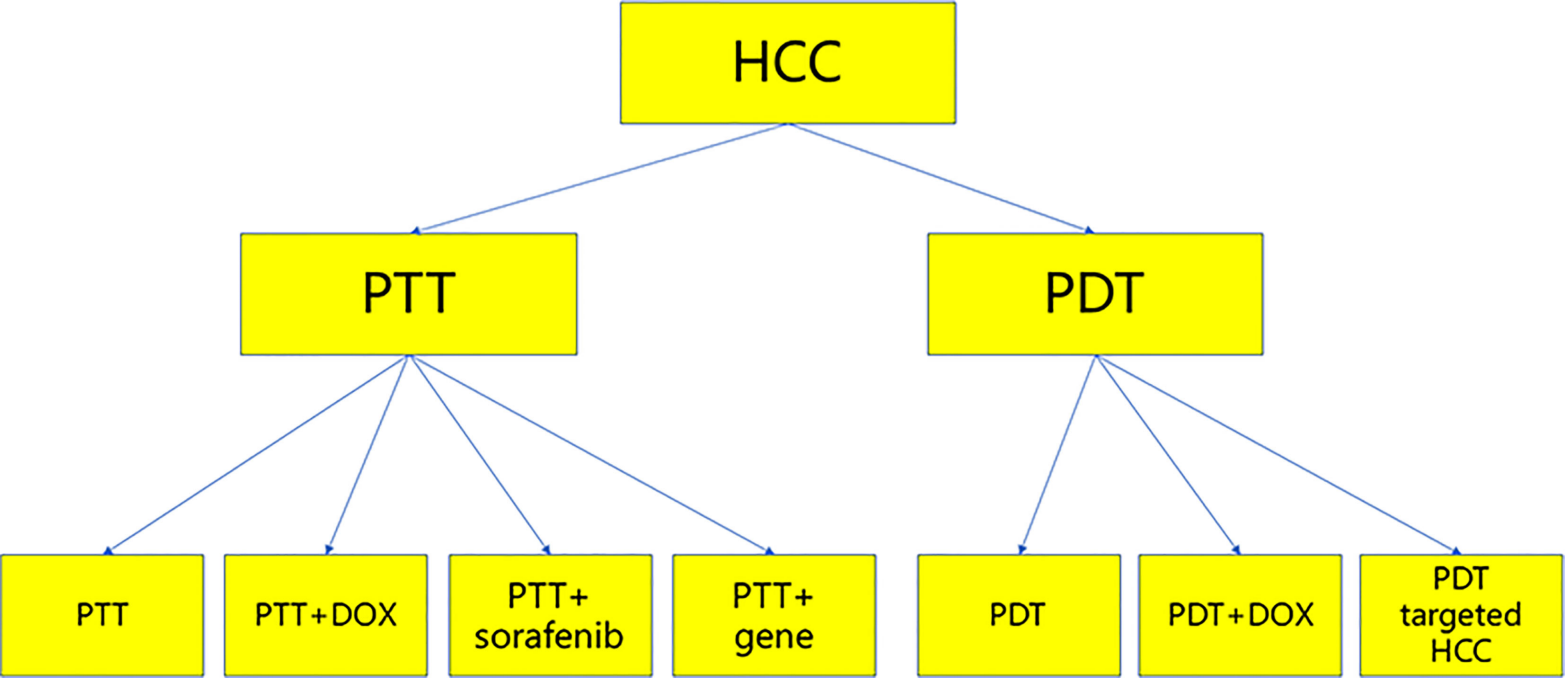
The summary charts.

PTT and PDT have played a significant role in the treatment of tumors, and they have been used to treat HCC. This paper reviews recent studies on the treatment of HCC by PDT and PTT, with the aim of exploring new ideas and strategies for the treatment of HCC (**Figure 2**).

## PTT

### PTT for HCC

The application of light to heat energy conversion in tumor diagnosis and treatment has attracted the extensive attention of researchers (18) (**Table 1**). Metal nanoparticles play an important role in the diagnosis and treatment of tumors (23). Strong near-infrared light absorption is the basis of metal nanoparticles in PTT. Compared with traditional treatment, metal nanoparticles have the characteristics of high selectivity and efficiency and they are minimally invasive (24). Gold nanomaterials are the most widely used (25) because they have tunable surface plasma resonance properties and strong photothermal conversion efficiency (26). Juan Hu et al. synthesized cubic gold nanoparticles with different sizes, which could be excited by near-infrared light at 808 nm wavelength, showed strong near-infrared light absorption, optical stability, photothermal effect and high biocompatibility and were effective for treating liver cancer cells and animal models (19).

**Table 1:** PTT for HCC.

Authors	Structure	Irradiation wave length
Juan Hu et al. (19)	Au-80 CNAs	808 nm
Huijing Xiang et al. (20)	Nano-BFF	1000–1700 nm
Weijie Ma et al. (21)	CAR-T-IM	808 nm
Jinghua Li et al. (22)	Bi@ZIF-8-gambogic acid(GBZ)	1064 nm
Hongqiao Cai et al. (12)	CuS-ATMi@TGF- βNPs	808 nm

The absorption wavelength of near-infrared light in zone I is 650–1000 nm, which has poor tissue penetration. The tissue penetration of near-infrared light in zone II is good, with a wavelength of 1000–1700 nm, but it is rarely used at present (27). Huijing Xiang et al. polymerized and self-assembled boron difluoride formazanate dye to turn it into a two zone near-infrared dye. *In vivo* and *in vitro* studies confirmed that the nanoparticles had deep tissue penetration of light in zone II and inhibited HCC (20).

Chimeric antigen receptor (CAR) T-cell therapy is an important emerging treatment for tumors. T cells of tumor patients are modified *in vitro* to carry tumor specific antigens, and then injected into patients to attack tumor cells (28). CAR-T-cell therapy has shown clinical efficacy and safety for hematological malignancies and solid tumors (29). CAR-T cells can specifically recognize tumor-associated antigens and eliminate tumor cells through the single chain variable region. This region is derived from the heavy and light chains of polyclonal antibodies and can be expressed on the cell membrane of CAR-T cells (30). Weijie Ma et al. synthesized mesoporous silicon with 2-[2-(2-chloro-3-[(1,3-dihydro-3,3-dimethyl-2h-1-propyl-indole-2-subunits) ethylidene]-1-cyclohexene-1-base) vinyl]-3,3-dimethyl-1-propyl indole Weng iodide.

After T cells were transfected with heparin sulfate proteoglycan-3 (GPC3)–CAR lentivirus, the cell membrane of T cells was separated to form the CAR-T capsule (CAR-Tc). Finally, the CAR-Tc and IM were assembled to form the CAR-Tc–IM, which showed a good photothermal effect on liver cancer cells and it killed HCC cells (21).

Traditional photothermal agents (PTAs) perform hyperthermia ablation *via* activation of near-infrared I region, but the penetration depth is not high. At the same time, the heat resistance caused by heat shock protein also restricts the therapeutic effect of PTT on tumors (31, 32). At present, the cost of PTAs used is often expensive. Bismuth is a classical semi-metallic element and a hot spot of scientific research (33, 34) because it is cheap and non-toxic (35). Most ZIF-8 nanodrug carriers are used for intravenous drug delivery, and are considered to be promising drug release and controlled release platforms (36). Jinghua Li et al. combined Bi and ZIF-8 through a one-step reduction method (Bi@ZIF-8), added gambogic acid to Bi@ZIF-8 to form Bi@ZIF-8–gambogic acid (GBZ), while gambogic acid could be an inhibitor of Hsp90. In addition to good biocompatibility, GBZ is important because the temperature of PTT is low, the damage to surrounding normal tissues is small and it has a good killing effect on HCC cells (22). Hongqiao Cai et al. noted the adverse effects of heat damage to normal tissues near tumors (11). They synthesized hollow structure CuS nanoparticles with ataxia telangiectasia mutated (ATM) inhibitor loaded with surface modified TGF-β antibody (CuS-ATMi@TGF- βNPs). The nanoparticles not only achieved low-temperature PTT, but also caused less damage to normal tissue, and had sufficient targeting and biocompatibility (12).

### PTT Plus Doxorubicin Treatment for HCC

PTT and chemotherapy often play a synergistic role. However, low targeting and poor drug delivery capacity are still the common shortcomings of photosensitizers and chemotherapeutic drugs. Therefore, it is of importance to design an effective nanodrug delivery platform to transport and control chemotherapeutic drugs and the accurate targeting of photothermal preparations (37, 38). Due to the depth of penetration, PTT often cannot eradicate tumors; therefore, PTT is often combined with chemotherapeutic drugs to achieve synergistic therapeutic effects and fewer adverse effects (39, 40). Doxorubicin is a classical chemotherapeutic drug with anthracycline structure. It has been used in the treatment of a variety of tumors, but there are many adverse effects, which affect its widespread application (41, 42).

The targeted and controlled release of drugs in the tumor area is the main difficulty in the treatment of HCC. In order to overcome this problem, Long Wu et al. designed a platelet cell membrane encapsulated polypyrole and doxorubicin nanoparticles (PLT PPy–DOX). These nanoparticles have photothermal activity because of PPy and chemotherapeutic activity because of doxorubicin. This platelet-simulated drug delivery system shows a good therapeutic effect on orthotopic HCC (43).

Polyethylene glycol (PEG), doxorubicin, mesoporous silica nanoparticles (MSNs) and CuS can be synthesized into nanoparticles (PEG–DOX–MSN@CuS nanoparticles), which have photothermal and chemotherapeutic effects on HCC. Specifically, CuS is irradiated by near-infrared, PTT can destroy MSNs, and then doxorubicin is released to kill HCC cells (44).

To focus on the anti-HCC effect and avoid adverse effects, Huili Li et al. synthesized PEG–hyaluronic acid (HA) 4–gold nanocages (AuNCs)–Dox (PEG–HA4–AuNCs–Dox) nanoparticles. PEG–HA4–AuNCs–Dox play the role of photosensitizers; doxorubicin is a classic chemotherapeutic drug; HA controls drug release into the tumor microenvironment; and PEG acts as a surfactant and increases the circulation time of nanoparticles (45).

Indocyanine green (ICG) has been approved by the US FDA for medical diagnosis and treatment (46). IR-820 is a cheaper analog of ICG (47). IR-820 and doxorubicin are hydrophilic molecules. For the treatment of liver cancer, their disadvantages are less circulating time in the body and rapid internal disappearance (48). Yue Jiang et al. has solved the above problems. Lactosylated IR-820 is assembled with doxorubicin to form LA-IR-820/DOX nanoparticles. Lactose IR-820 has the characteristics of liver cancer targeting and photosensitizer (49), and doxorubicin can lead to immune cell death and consolidate the effect of PTT (50).

Multidrug resistance (MDR) occurs in the treatment of various tumors and is a major challenge in tumor treatment (51). P-glycoprotein (P-gp) is overexpressed in many MDR cell lines, resulting in an increase of MDR (52).

Weiping Wang et al. found that anti-mir-21 can effectively inhibit P-gp and upregulate expression of PTEN to enhance sensitivity to chemotherapeutic drugs. Therefore, a novel nanoparticle system was synthesized, HA/anti-miR-21/PPAuNCs (HA-conjugated, anti-miR-21-loaded, PEI-modified PEGylated AuNCs). In addition to enhancing the sensitivity of HepG2/ADR cell line to chemotherapy, AuNCs can also play the role of PTT by mild near-infrared irradiation (53).

The 5-year recurrence rate of patients with liver cancer is 70–80%, which urgently needs to be resolved. Theoretically, the treatment of recurrent liver cancer is repeat hepatectomy or liver transplantation. The results of repeat hepatectomy, transarterial chemoembolization and radiofrequency ablation are poor (54). The combination of PTT and chemotherapy has an obvious synergistic antitumor effect (55). In order to treat recurrent liver cancer by PTT and chemotherapy, a homotypic tumor cell membrane drug delivery platform thermosensitive liposome–HCC cell membrane (HepM–TSL) was synthesized. This platform consists of thermosensitive liposome vesicles and HCC cell membranes, and ICG and doxorubicin are encapsulated by the above platform (ICG–DOX–HepM–TSL). ICG–DOX–HepM–TSL can avoid the immune system and directly target recurrent HCC. Excitation at 808 nm can lead to the decomposition of TSL, and the photothermal and chemotherapeutic effects of ICG and doxorubicin can be realized. At the same time, this platform also has good therapeutic effects and few adverse effects (56).

Tumor thermal ablation has become an effective method for local treatment of HCC, but it is not recommended for HCC with local recurrence > 3 cm (57). MoS_2_ has become an ideal PTT reagent because of its excellent surface plasmon resonance characteristics, photothermal conversion efficiency and biocompatibility (58). 300 nm diameter hollow MoS2 nanoparticles were established, and then doxorubicin was embedded (DOX@MoS2). The antitumor effect of the nanoparticles was confirmed by *in vitro* and *in vivo* experiments (59).

### PTT Plus Sorafenib Treatment for HCC

Sorafenib, a type of multikinase inhibitor, is the first-line drug treatment for advanced HCC approved by the United States FDA (60). However, sorafenib’s disadvantages include poor drug targeting and poor water solubility of oral sorafenib (61). With the emergence of nanotechnology, sorafenib has become more effective for treatment of liver cancer.

Tianjun Zhou et al. designed nanoparticles of SP94–PB–SF–Cy5.5, which included sorafenib (SF), Prussian blue porous metal organic frame (PB), HCC-specific targeting peptide SP94, and near-infrared dye cyanine 5.5 (Cy5.5) (62). PB is an FDA-approved drug for thallium poisoning (63). It can be designed as a metal organic framework to carry drugs and combine with fluorescent dyes (64). Sp94 is an HCC-specific targeting polypeptide that can achieve specific binding between nanoparticles and HCC cells. Cy5.5 is a near-infrared dye that can be excited by 808 nm visible light (65). SP94–PB–SF–Cy5.5 achieved no recurrence of HCC in a HepG2 cell line nude mouse liver cancer model (62).

A macrophage–cancer cell membrane hybrid has been constructed. The membrane packages hollow CuS nanoparticles that contain sorafenib; and the membrane is surface modified with anti-VEGFR antibodies (CuS-SF@CMV NPs). The anti-VEGFR antibody can kill tumor cells by inhibiting angiogenesis *via* PI3K/AKT pathways. The nanoparticles avoid the immune system through immune escape, accurately locate HCC cells through liver cancer targeting, and kill tumor cells through PTT and kinase inhibition (66, 67).

### PTT Plus Gene Therapy for HCC

MSNs are widely used because of their high specific surface area, controllable shape and easy surface functionalization (68, 69). Silica nanoparticles have a sharp surface, which has strong plasmid DNA binding ability and transfection performance (70). Mesoporous silica nanoparticles (MSNs) and Au NR core can be synthesized into Au@MSNs, and addition of PEG forms Au@MSN–PGEA. Au@MSN–PGEA, SF, and P53 gene can be synthesized into Au@MSN–PGEA@SF@P53 nanoparticles. besides PTT and targeted therapy, Au@MSN–PGEA@SF@P53 nanoparticles also increase the role of gene therapy for HCC (11).

## PDT

### PDT for HCC

PDT has been widely used for cancer. During PDT, reactive oxygen species (ROS) are generated, such as singlet oxygen, that can damage cancer cells (71). The principle of PDT is that a photosensitizer is excited by a specific excitation wavelength of light, converts energy into oxygen molecules in cell to form ROS, and ROS act on tumor cells (72), which can directly induce cell death, disturb tumor vasculature and activate the innate immune system (73).

As a second-generation photosensitizer, Radachlorin has a strong absorption band at 662 nm and has excellent physical and chemical properties, such as weak dark toxicity and rapid *in vivo* metabolic rate (74). Hamidreza Mirzaei et al. found that Radachlorin can induce HepG2 cell apoptosis through PDT, but it has no obvious harmful effect on HFLF-PI4 cells (75).

Metal phthalocyanines are photosensitizers that have been used in the treatment of tumors. Jingwei Shao et al. synthesized photocyanine and a series of analogs: tetra-triethyleneoxysulfonyl zinc phthalocyanines (ZnPcs). When photocyanine is activated by 670 nm excitation, it promotes apoptosis and necrosis of HepG2 cells by producing ROS, activating caspase-3 and stagnating cells in G2/M phase (76). ZnPc is also used in PDT of HCC cells. It can inhibit mitogen-activated protein kinase and extracellular signal-regulated kinase pathways through PDT, and upregulate Bax and downregulate Bcl-2 to destroy cancer cells (77). Gold nanoparticles combined with photosensitizer can be used for PDT of liver cancer cells. Pu-18-N–butylimide–N-methyl-D-glucamine (NMGA) is a new photosensitizer that combines with gold nanoparticles to form Pu-18-N–butylimide–NMGA–GNP. It can significantly reduce transplanted liver cancer under excitation light of 640–710 nm (78). Lactosomes are core-shell nanoparticles including amphiphilic polymeric micelles. ICG lactosomes were injected into male BALB/c nude mice through the caudal vein for 48 h. After xenograft tumors were stimulated by near infrared laser (AVL-15), a large number of apoptotic tumor cells could be observed (79).

Tumor tissue is different from non-tumor tissue in many biological and chemical aspects, and the tumor microenvironment is more likely to be acidic (pH 6.5–6.8) (80); therefore, an acidic environment is often used for activation of pH-responsive photosensitizer (81). However, the acidic activation pH of most pH-responsive photosensitizers is < 6 (82), which means that not all photosensitizers are pH responsive. Some photosensitizers can obtain pH-responsive function through being modified by pH-responsive groups, such as phthalocyanine dimer modified by an acid-sensitive unit (83), polysaccharide/Ce6 conjugate modified by pH-induced functional group (84) and cyclometalated iridium (III) complexes modified by benzimidazole (85). The activation efficiency of the above photosensitizers is not high, which limits their application (86). Layered double hydroxides (LDHs) have attracted much attention because of their ability to carry drugs or genes, as well as acid sensitivity and anion exchange properties (87, 88). ZnPcS_8_ has high photosensitivity efficiency, but it has the shortcomings of aggregation and rapid metabolism in the body. In order to overcome these shortcomings, Xingshu Li et al. synthesized LDH–ZnPcS_8_. The pH response of LDH–ZnPcS_8_ is reflected in high quenching effects at pH 7.4 and high reactivating effects at pH 6.5. There were strong PDT effects on HepG2 cells with LDH–ZnPcS_8_ at pH 6.0/6.5 compared with at pH 7.4 (86).

Metal-organic frameworks (MOFs) have been used for PDT research on tumor cells. Due to the low-oxygen environment in tumor cells, MOFs are not efficient at converting oxygen molecules in tumors into singlet oxygen. Platinum nanozymes can be decorated to MOFs to form high catalase-like activity that could produce a more efficient PDT effect (89).

### PDT Plus Doxorubicin Treatment for HCC

Doxorubicin is a classic chemotherapeutic drug that has been used in the treatment of many types of tumors, but its adverse effects are serious and affect its application (90). Sulfonated aluminum phthalocyanine (AlPcS) has the following characteristics: good water solubility, strong absorption band in the red light region, and high singlet oxygen output rate (91). However, the sulfonated group in AlPcS reduces the affinity of AlPcS for the cell membrane (92). AlPcS–DOX conjugates can increase the uptake of AlPcS by HCC cells (hepatology cell line 7701), doxorubicin can act on the DNA of HCC cells, and AlPcS-mediated PDT targets lysosomes to kill HCC cells (93).

### PDT Targeted HCC

Mitoxantrone, a type II topoisomerase inhibitor, is an antitumor drug (94). At the same time, it is also an efficient photosensitizer with two major absorption peaks at 610 and 660 nm (95). Epithelial cell adhesion molecule (EpCAM) is considered to be an important marker of cancer stem cells (96), and is associated with poor outcomes of HCC (97). Yong Han et al. grafted mitoxantrone with anti-EpCAM antibody to synthesize anti-EpCAM nano-micelles, which can recognize the EpCAM of HCC cells and have targeting properties, and then mitoxantrone exhibits excellent chemotherapeutic and PDT effects (95).

Folate receptor (FR) expression is lower in normal cells but higher in tumor cells. Folic acid (FA) can bind to its specific receptor (98). Porphyrin MOFs consist of porphyrin and metal ions, and have excellent biocompatibility and good dispersibility, as well as being effective for PDT (99). Gd-MOFs are synthesized in combination with FA. These nanoparticles can be recognized by fluorescence and magnetic resonance imaging, and can specifically target FR-positive cancer cells. Once inside the cell, the effect of PDT is highlighted (100).

Integrin αvβ3 is an angiogenesis driver in malignant tumors, and plays an important role in HCC (101). A hydrophilic and targeted peptide (cRGD) can be recognized by integrin αvβ3 *via* receptor-mediated endocytosis (102). Fluorogens with aggregation-induced emission (AIE) have been used in biotechnology. Fluorogen derivatives with AIE (TPETS nanodots) can be used to treat cancer cells and ROS are generated by visible light irradiation (103). Yang Gao et al. modified cRGD on TPETS nanodots, which had the ability to target cRGD to be recognized by integrin αvβ3, but also has a PDT effect on HCC cells (104–106).

## Conclusion and Future Prospects

HCC is a malignant tumor with poor prognosis and high mortality, and is difficult to detect in the early stage, which seriously endangers human health. Research efforts have focused on finding an effective treatment. Over the years, surgical treatment and chemotherapy, as well as the current emerging targeted therapies and immunotherapy, have been shown to have therapeutic effects on HCC. In recent years, the combination of medical and engineering methods as a treatment strategy for liver cancer began to achieve results. However, most of the current studies are based on basic research, and there are still few clinical PTT- or PDT-based HCC studies. Maybe there are good strategies combining immunotherapy/targeted therapy with PTT/PDT; at the same time, accelerating the transformation of basic research into clinical research and the promotion of clinical research into clinical application are effective approaches for the treatment of HCC, With the development of science and technology and the deepening of research, effective treatment of liver cancer will improve.

## Author Contributions

ZF, CZ, SW, and YZ contributed to conception and design of the article. ZF organized the database. ZF wrote the first draft of the manuscript. CZ and SW wrote sections of the manuscript. YZ revised the manuscript. All authors contributed to manuscript revision, read, and approved the submitted version.

## Funding

This study received financial support from the National Natural Science Foundation of China (NO. 81701965, 81872255); Natural Science Foundation of Liaoning Province (NO. 20180550116, 2019-MS-069); Doctoral Research Initiation Fund of Liaoning Province (2020-BS-187).

## Conflict of Interest

The authors declare that the research was conducted in the absence of any commercial or financial relationships that could be construed as a potential conflict of interest.

## Publisher’s Note

All claims expressed in this article are solely those of the authors and do not necessarily represent those of their affiliated organizations, or those of the publisher, the editors and the reviewers. Any product that may be evaluated in this article, or claim that may be made by its manufacturer, is not guaranteed or endorsed by the publisher.
